# Factors affecting hospital inpatient blood pressure measurement as ranked by a Delphi survey

**DOI:** 10.1038/s41598-026-46429-6

**Published:** 2026-04-05

**Authors:** Alexandra Gallagher, Brendan Smyth, George Mangos, Aletta E. Schutte, James E. Sharman, Meg J. Jardine, Mark Brown, Inpatient Blood Pressure Measurement Expert Panel, George Mangos, Aletta E. Schutte, James E. Sharman, Mark Brown, L. Burrell, S. Hiremath, S. Kotwal, A. Levin, A. Makris

**Affiliations:** 1 NHMRC Clinical Trials Centre, University of Sydney, Camperdown, Australia; 2 Department of Renal Medicine, St George Hospital, Kogarah, Australia; 3 School of Clinical Medicine, UNSW Medicine & Health St George & Sutherland Clinical Campus, Sydney, NSW Australia; 4 School of Population Health, University of New South Wales, Sydney, NSW Australia; 5 The George Institute for Global Health, Sydney, Australia; 6 Menzies Institute for Medical Research, University of Tasmania, Hobart, Australia; 7 Department of Renal Medicine, Concord Repatriation General Hospital, Sydney, Australia; 8 Department of Medicine, University of Melbourne, Austin Health, Melbourne, Australia; 9 University of Ottawa, Ottawa, ON Canada; 10 Department of Nephrology, Prince of Wales Hospital, Sydney, NSW Australia; 11 Division of Nephrology, University of British Columbia, Vancouver, Canada; 12 School of Medicine, Western Sydney University, Penrith, Australia

**Keywords:** Inpatient, Blood pressure measurement, Delphi survey, Cardiology, Health care, Medical research

## Abstract

Current guidelines do not address the appropriate technique for accurate inpatient blood pressure (BP) measurement. Outpatient data suggest that technique matters for accurate readings. Given the substantial impact of BP treatment decisions, this study explored the relative importance of various factors for accurate inpatient BP measurement. This was a ranking-type Delphi survey conducted in two stages. Stage 1 involved document ratification (single round) where an international panel of 9 hypertension experts reviewed and validated source documents relevant to BP measurement. Stage 2 involved variable ranking across three iterative rounds: Rounds 1 and 2 required panellists to independently rank all 10 variables from most to least important with each variable receiving a unique rank; Round 3 used paired comparisons with forced choices to refine consensus. Binary decisions required > 80% agreement for consensus. Ranked order consensus required Kendall’s coefficient > 0.7. The panel agreed with high consensus on the following 10 variables in order of importance: (1) at least two measurements, (2) correct cuff size, (3) not talking during measurement, (4) correctly fitted cuff, (5) cuff at heart level, (6) seated position, (7) contemporary device calibration, (8) legs uncrossed, (9) bare arm below cuff, and (10) supported back (Kendall’s 0.97, P < 0.001). Understanding of true determinants of accurate inpatient BP measurement is still required. Meanwhile, expert consensus provides an intermediary step for prioritising factors in inpatient BP measurement to assess performance.

**Supplementary Information:**

The online version contains supplementary material available at 10.1038/s41598-026-46429-6.

## Introduction

High blood pressure (BP) is common and the greatest risk factor for cardiovascular disease globally^1^ Lowering BP is proven to lower the risk of cardiovascular and kidney complications of high BP^2^ Successful BP diagnosis and management requires accurate measurement. To this effect, collaborative efforts have established evidence-based guidelines for the accurate measurement of BP in office and community settings^3–7^ Elevated BP in hospital is common also, but its determinants and reason for assessment differ from the outpatient context^8^ Inpatient BP is measured primarily to detect acute clinical deterioration, and individuals are not necessarily at their steady state^8^ It is also unclear if lowering high BPs in this population is of benefit in the short or long term and some growing evidence suggests that intensifying chronic management may also lead to harm, especially in older adults^9,10^.

As opposed to the outpatient setting, there are no guidelines on how to accurately measure BP in the inpatient setting. This is often reported by leading BP societies to be a result of insufficient empirical evidence, and an area of recommended extended research^3–7^ As with other areas of limited evidence in healthcare, inpatient BP measurement practices default to adopting models from similar clinical settings, in this case the office and community, until such time that the evidence gap can be bridged. A more rigorous intermediary step is to apply expert opinion to determine the suitability of this adaptation of guidelines.

The Delphi method to reach consensus is a systematic approach for gathering and refining insights from a group of experts^11–13^ The Delphi method is widely regarded as an effective way to achieve consensus on topics that lack substantial empirical evidence^14^ In light of the known dearth of empirical evidence on BP measurement in the hospital setting, this method of determining consensus was deemed the most appropriate.

This study aimed to establish a hierarchy of the key procedural factors for accurate inpatient BP measurement through expert consensus. By identifying and ranking these essential variables, we aimed to create the foundation for a standardized quality assurance framework that can transform inconsistent BP measurement practices across clinical settings, assuring more precise readings for clinical interpretation.

## Methods

The study was conducted in alignment with the ACCORD (ACcurate COnsensus Reporting Document) and EQUATOR Network guidance for reporting consensus-based methods in biomedical research^14^ These guidelines justified the use of Delphi methodology over other methods as the most appropriate for the study aims and context. This survey was conducted as the systematic consultation of expert opinion from a key group of leaders in the field and did not collect data on their clinical practice, personal views, or experience. Informed written consent from each panellist was obtained via email prior to participation in the survey. Anonymity of opinions during this survey and the absence of sensitive data collection was assured. As such it represents a form of consensus guideline development rather than research on human subjects and so was exempt from the need for ethical review.

### Guideline review

**Fig. 1:**
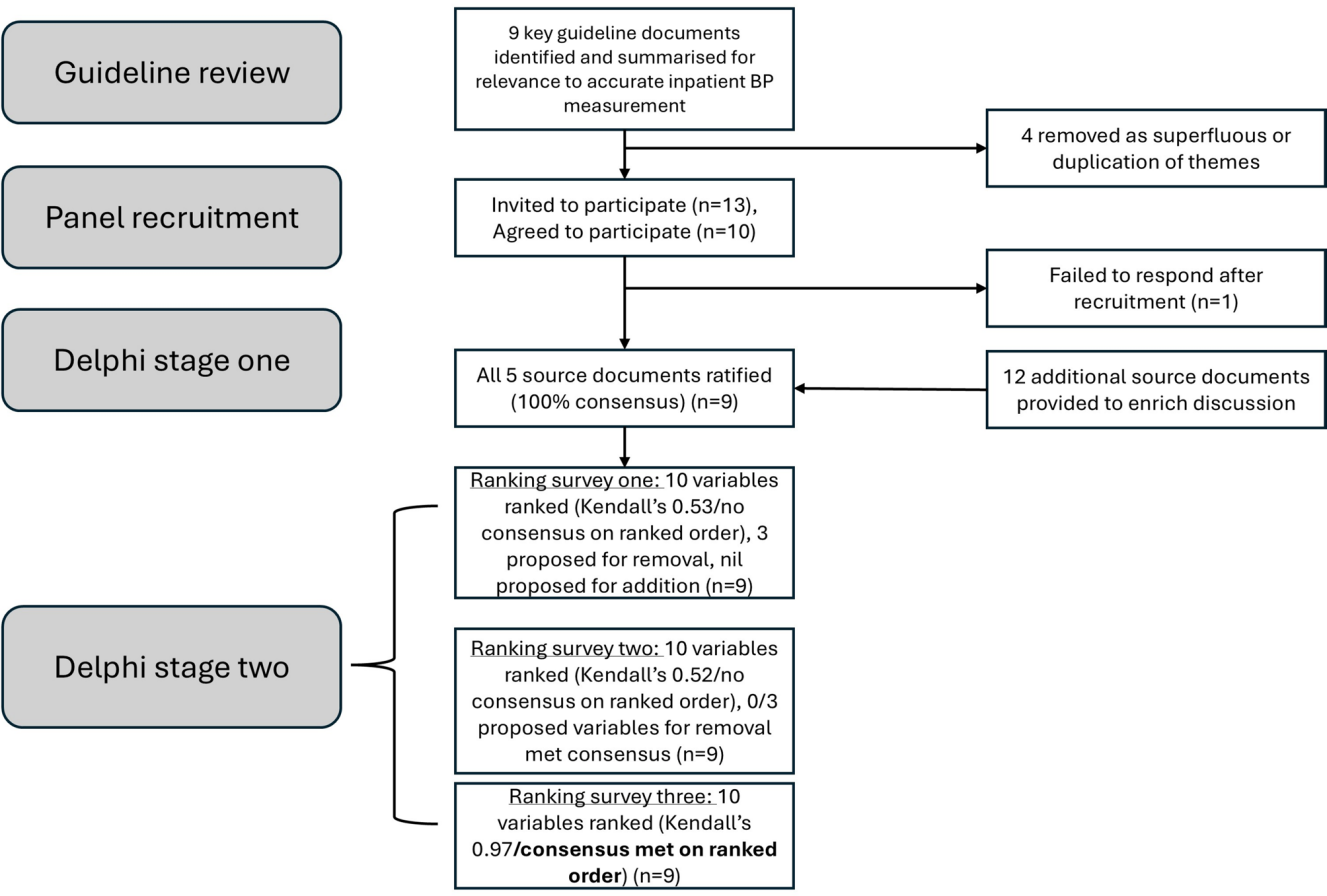
Flow diagram of study.

A review was performed to identify what evidence existed (1) to guide accurate measurement of BP in general, regardless of environmental setting, (2) to guide accurate measurement of BP in the inpatient setting and (3) to determine which aspects of BP measurement had the greatest magnitude of effect on accuracy. This involved a review of international BP guidelines and their referenced evidence base (see Supplementary Table 1 and Fig. 1 for the detailed search strategy).

Seven fundamental guidelines were identified detailing the procedural steps for accurate BP measurement (Table 1). Two additional papers were acquired from the referenced works were thought appropriate for specific mention. The first was the 2021 European Society of Hypertension (ESH) practice guidelines for office and out-of-office BP measurement^15^ as this document was referenced without alteration by the current 2023 ESH Guidelines in the instructions for BP measurement^5^ The second was a systematic review used by all guidelines as the summative resource on the factors that impact the accuracy of BP measurement^16^ A third paper was also considered, the American Heart Association (AHA) scientific statement detailing the management of elevated BP in the acute care setting which seemed pertinent given its specificity to hospitalised patients^17^.

**Table 1:** Comparative summary of 11 categories of conditions for correct blood pressure measurement as per current international guidelines.

Condition	2017 ACC/AHA Guidelines^6^	2019 AHA scientific statement on BP measurement^3^	2020 ISH Guidelines^20^	2020 WHO technical specifications for BP measuring devices^19^	2023 ESH Guidelines^5^^	2023 International consensus on standardised BP measurement^4^	2024 ESC Guidelines for the management of elevated BP and HTN^7^
Rest period	5 min	3–5 min	3–5 min	5 min	3–5 min	3–5 min	5 min
Position	Seated, back supported, feet flat on floor	Seated or supine, back supported, legs uncrossed, feet flat on floor	Seated, back supported, feet flat on floor	Seated, back supported, feet flat on floor	Seated, back supported, feet flat on floor	Seated, back supported, feet flat on floor	Seated, back supported, legs uncrossed
Arm position	Supported at heart level	Supported at heart level	Supported at heart level	Supported at heart level	Supported at heart level	Supported at heart level	Supported
Cuff size	Appropriate for arm circumference	Appropriate for arm circumference	Appropriate for arm circumference	Appropriate for arm circumference	Appropriate for arm circumference	Appropriate for arm circumference	Appropriate for arm circumference
Cuff position	Cuff bladder sufficiently encircles upper arm	Cuff bladder sufficiently encircles upper arm, lower end of cuff 2–3 cm above the antecubital fossa and firmly fitted	Cuff bladder sufficiently encircles upper arm	Cuff bladder sufficiently encircles upper arm, lower end of cuff 2–3 cm above the antecubital fossa and firmly fitted	Cuff bladder sufficiently encircles upper arm, lower end of cuff 2–3 cm above the antecubital fossa and firmly fitted	Cuff ends 2–3 cm above the antecubital fossa	Bladder length 75–100% and the width 35–50% of the arm circumference a few cm from antecubital fossa
Number of measurements	At least 2, 1–2 min apart	At least 2, 1–2 min apart	3 readings, 1 min apart, averaging second 2	At least 2, 1–2 min apart	3 readings, (2 if normal), averaging the second 2, 1 min apart	At least 2 readings, at least 30 s apart	3 readings, averaging the second 2, 1–2 min apart. Obtain further if readings differ by > 10 mmHg
Caffeine and smoking abstinence	30 min	30 min	30 min	30 min	30 min	30 min	30 min
Bladder emptying	Before measurement	Before measurement	Before measurement	Before measurement	Not specified	Before measurement	Before measurement
Talking during measurement	Not allowed	Not allowed	Not allowed	Not allowed	Not allowed	Not allowed	Not specified
Room temperature	Comfortable	Not specified	Comfortable	Comfortable	Comfortable	Comfortable	Not specified
Clothing on arm	Bare arm	Bare arm	Bare arm	Bare or light sleeve acceptable	Bare arm	Bare arm	Bare arm
Device validation	Validated device	Validated device	Validated device	Validated device	Validated device	Validated device	Validated device
Device calibration	“Periodically calibrated”	“Regular recalibration”	“Appropriately calibrated”	1–2 yearly calibration, monthly surveillance of tubing and cuff	Annual calibration with focus on integrity of tubing and cuff	Not specified	Not specified

*BP *Blood pressure, *HTN *Hypertension, *ACC *American College of Cardiology, *AHA *American Heart Association, *ISH *International Society of Hypertension, *WHO *World Health Organisation, *ESH* European Society of Hypertension ^Reference 2021 ESH guidelines for office and out-of-office BP measurement^15.^

A 2017 systematic review detailed the pooled results of 328 empirical studies relevant to the measurement of adult’s resting BP with specific reference to the potential sources of inaccuracy^16^ It attempted to quantify the effects of 29 potential sources of inaccuracy on measured BP values. Both the authors of the original review and those of the present study made note of the varying magnitude of effect, bidirectionality of single effects and the potential for multiple variables to create an incalculable net effect.

The systematic review was used to weight the variables identified by the 7 BP guidelines as essential components of accurate BP measurement for importance on BP accuracy, resulting in Supplementary Table 2. The findings of the 2024 AHA statement on the management of elevated BP in the acute care setting^17^ were used to narrow the list of variables for the inpatient setting. This AHA statement reinforced the topic of accurate inpatient BP measurement was an area of importance, with a great degree of variation in practice and also that the preferred measurement technique, other than to model practices on outpatient standards, was beyond the scope of their current statement, thereby validating the focus of our paper^17^.

A pragmatic adaptation of the outpatient guidelines for BP assessment was generated, identifying the key procedural variables for adult BP measurement. Prior to Delphi panel review, the research team evaluated outpatient BP measurement variables for feasibility in the inpatient setting. Variables such as extended rest periods, guaranteed quiet environments, and deferral of measurement due to physiological complaints such as pain or anxiety were excluded as they are often impractical or inappropriate in acute care where timely vital sign monitoring is essential for patient safety. The study focused on controllable measurement technique variables that could be standardized without compromising clinical workflow. A shortlist of these key documents was compiled for ratification by expert panel (Supplementary Table 3).

### Delphi method

The Delphi technique is based on three main principles: anonymity during voting, which ensures participants can express their views without their identities influencing decision making; iterative rounds of rating ideas, allowing for refinement and reconsideration; and controlled feedback between rounds, which provides structured information to guide responses in subsequent rounds^11^. The ranking-type Delphi method is a subtype that seeks agreement on the relative importance of a set of variables^11^.

#### Panel selection and characteristics

To meet criteria as an expert for this panel, respondents needed to: (1) participate in research in the field of BP, (2) hold leadership positions in the field, and (3) be involved in the development of guidelines for the diagnosis, management or treatment of hypertension. For clinicians, specialist training in BP management or direct involvement in inpatient BP management were required. For researchers, a demonstrated focus on BP and cardiovascular health research was required, determined by review of their academic portfolio.

A minimum of eight panellists was deemed necessary based on extant literature^11^, with efforts made to ensure adequate gender diversity and geographic representation. Participants were recruited via email invitation from author lists of publications relevant to the field. Ten clinician researchers were initially invited on July 8, 2024. Three declined, and an additional three experts were subsequently invited, with two consenting to participate.

The expert panel comprised of nine panellists (Supplementary Table 4): five females and four males, based in Canada (Ottawa and British Columbia) and Australia (New South Wales, Victoria and Tasmania). Three of the panellists were dedicated researchers (L.B, A.S and J.S) while six were clinician researchers, all clinical nephrologists. All panellists had participated in the creation of hypertension guidelines, cardiovascular/BP research, with a cumulative count exceeding 2,300 articles.

#### Delphi survey design

This Delphi survey was designed in accordance with the CREDES^12^ and ACCORD^14^ recommendations and comprised of two sequential stages:

Stage 1: Document Ratification (single round). Panellists reviewed source documents from the literature review to validate their appropriateness for identifying variables affecting inpatient BP measurement accuracy. This stage included open ended questions, opportunities for comments, and provision of additional source documents. Consensus required > 80% agreement on the suitability of provided documents. Anonymised feedback was provided prior to progressing to stage two.

Stage 2: Variable Ranking (three iterative rounds). Panellists completed anonymised surveys using Qualtrics_®_ software, ranking variables affecting inpatient BP measurement accuracy from 1(most important) to 10 (least important). A maximum of 3 rounds were planned to avoid respondent fatigue^18^.

*Round 1:* Panellists independently ranked all 10 variables from 1 (most important) to 10 (least important) with each variable required to receive a unique rank (no tied rankings permitted). Panellists could also recommend additions or deletions to the variable list (requiring > 80% agreement for implementation). No variables met the threshold for modification.

*Round 2:* Following feedback consisting of mean ranks and de-identified comments from Round 1, panellists re-ranked all variables from 1 (most important) to 10 (least important), again with each variable required to receive a unique rank.

*Round 3:* To refine consensus and reduce residual disagreement, panellists were presented with five paired comparisons derived from their individual Round 2 rankings. Each panellist’s Round 2 ranking was used to create five pairs: the variables they ranked 1 st and 2nd, 3rd and 4th, 5th and 6th, 7th and 8th, and 9th and 10th. For each pair, panellists made a forced choice, selecting which variable was more important for BP measurement accuracy (Appendix Document D). This pairwise forced-choice method facilitated final rank stabilization.

Consensus criteria: Consensus on the ranked order of variables was measured using the Kendall’s W coefficient of concordance, which determines agreement among multiple rankings and ranges from 0 (no agreement) to 1 (complete agreement). A Kendall’s coefficient of ≥ 0.7 was defined a priori as indicating very strong agreement and meeting the threshold for consensus. Confidence intervals for Kendall’s W were not calculated as is standard practice in Delphi consensus studies, where the focus is on the magnitude of agreement (W coefficient) and its statistical significance (p-value) rather than precision estimation.

## Results

### Guideline review

The included guidelines were more or less in accord on conditions for an accurate BP measurement (Table 1). Indeed, any discrepancies were mostly that of omission, such as in the description of the correct cuff position and what was deemed appropriate for the frequency of device calibration. A correct BP measurement technique was thought to require thirteen conditions, dictating the adequate rest period, positioning and preparation of the patient, cuff characteristics, number of measurements, ambient conditions and technical specifications for the device used. The noticeable variations between guidelines were the permissibility of a light sleeve below the cuff by the World Health Organisation (WHO) and supine position by the AHA. The International Society of Hypertension (ISH) were also explicit in requiring a total of three BP measurements, whereas the other guidelines supported a conditional minimum of two. Calibration was recommended to match the manufacturer’s recommended interval of maintenance, with specific focus on cuff and tubing integrity. All guidelines advised use of appropriately validated devices to ensure accurate readings.

The result of this guideline review was the proposed list of ten variables for expert panel review during the Delphi survey (Table 2). This list excluded parameters that are unavoidable in the inpatient setting (for example a quiet environment) and prioritised what the guidelines and empirical evidence deemed of importance for accuracy.

**Table 2:** Overall rank, mean rank and Kendall’s coefficient of agreement for each round of Stage 2 variable ranking (Rounds 1–3).

Rank	Round 1 (mean rank [SD])	Round 2 (mean rank [SD])	Round 3 (mean rank [SD])
1	Correct cuff size (2.778 [1.8])	At least two BP measurements taken (2.333 [2.2]))	At least two BP measurements taken (1.444 [0.5])
2	At least two BP measurements taken (2.889 [2.0])	Correct cuff size (2.667 [1.8])	Correct cuff size (1.556 [0.5])
3	Not talking during BP measurement (3.889 [3.0])	Not talking during BP measurement (3.667 [3.2])	Not talking during BP measurement (3.444 [0.5])
4	Cuff at heart level (4.556 [2.7]) AND Patient in seated position (4.556 [1.6])	Cuff correctly positioned/fitted to upper arm (4.778 [2.2])	Cuff correctly positioned/fitted to upper arm (3.556 [0.5])
5	N/A	Patient in seated position (5.444 [1.4])	Cuff at heart level (5.222 [0.4])
6	Cuff correctly positioned/fitted to upper arm (5.111 [2.0])	Cuff at heart level (5.556 [2.8])	Patient in seated position (5.778 [0.4])
7	Device calibrated within recommended period as per manufacturer of device (6.667 [2.7])	Legs uncrossed (6.889 [1.9]) AND Device calibrated within recommended period as per manufacturer of device (6.889 [2.2])	Device calibrated within recommended period as per manufacturer of device (7.444 [0.5])
8	Legs uncrossed (7.111 [1.8])	N/A	Legs uncrossed (7.556 [0.5])
9	Arm bare below cuff (8.333 [1.5])	Arm bare below cuff (8.333 [1.5])	Arm bare below cuff (9.444 [0.5])
10	Back supported (9.111 [0.8])	Back supported (8.444 [0.8])	Back supported (9.556 [0.5])
Kendall’s coefficient	0.53 (P < 0.001)	0.52 (P < 0.001)	0.97* (P < 0.001)

*Consensus met, SD standard deviation.

### Delphi study

There was a 100% response rate from panellists for all rounds of the study (Stage 1 document ratification and all three rounds of Stage 2 variable ranking). Of note, the four co-authors that served on the expert panel remained blinded to the identities of the panel members, their responses and details of the Delphi process (GM, AS, JS, MB) until consensus was reached.

### Stage one: document ratification

The 9 panellists received the first survey via email on the 19^th^ of July 2024 (Supplementary Document A). There was 100% consensus on the appropriateness of the 4 key source documents, taken as representative guidelines for BP measurement and Kallioinen et al.’s systematic review to guide subsequent stages of the survey^3,4,16,17,19^ (Supplementary Table 2). Panellists were oriented to make these considerations with specific reference to the inpatient population and provide additional feedback and resources if desired (Supplementary Tables 5 and 6, respectively).

While the expert panel unanimously affirmed the four selected documents, they encouraged recognition of the absence of representation of pregnant women and children in the source documents and therefore an emphasis on the output of this survey not extending its recommendations to these special populations. There was strong support for the systematic review as the ideal source to inform later survey responses. The Kallioinen et al. review was described as “detailed”, “thorough” and “unique” in its consideration of bi-directional effects of sources of inaccuracy and summation of the available literature. The panel advised caution in its application to the inpatient setting, as the primary studies did not explicitly include hospitalised populations, and the quality of the empirical data and its analysis had limitations in a qualitative sense.

The recommended additional resources from the panel, included 2 guidelines that were identified in the initial guideline review by this paper’s authors^6,20^ These were recommended for their additional depth of appraisal of BP measurement methodology and role as a more international guideline. Particular emphasis was also placed on the importance of a validated device and technique for accurate measurement with 4 additional resources provided in support of appropriate methods and emphasis on the impacts of validation, one of which was already highlighted in the initial guideline review (Supplementary Table 6)^21–24^.

A further four supplied resources from the expert panel detailed additional empirical evidence for the magnitude of effects cuff size, rest periods, arm position and, arm circumference have in the procedure of BP measurement can have on accurate BP reading^25–28^ These were supportive of the Kallioinen review findings, rather than contradictory. A final additional resource detailed the protocol for and active randomised control trial examining the diagnostic accuracy of an inpatient BP in detecting chronic hypertension^29^ As the results of this study are not yet available, they were not able to inform this study.

### Stage 2: variable ranking (three iterative rounds)

**Fig. 2:**
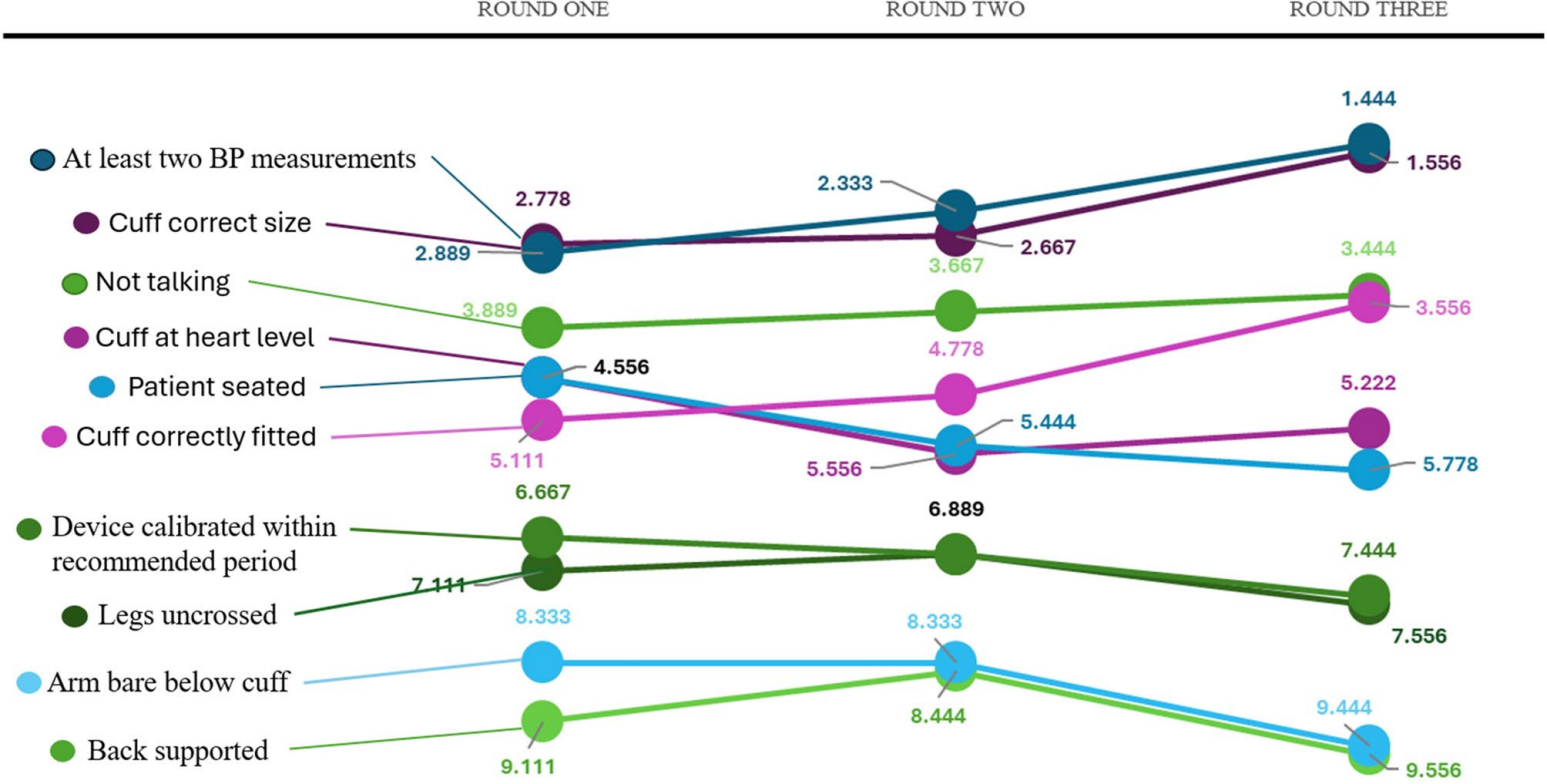
Slope graph demonstrating variation in ranked order of variables over three rounds of Stage 2 variable ranking.

Panellists required a total of three ranking survey rounds to reach consensus on the order and composition of the variables (Table 2 and Fig. 2). The construct of each anonymised Qualtrics based survey is available in the appendix (Documents B-D).

Round 1: Panellists independently ranked all 10 variables from 1 (most important) to 10 (least important), with each variable required to receive a unique rank. During this round, three of the ten variables were proffered for removal from the list as not necessary for an accurate BP reading: a bare arm below the BP cuff, a supported back and uncrossed legs. None of these met the required > 80% consensus for removal from the list during the subsequent round of the survey (Table 3).

**Table 3:** Panel review of proposed removal of non-essential components of accurate inpatient blood pressure measurement.

Variable	Proportion of panel proposing removal	Comments	Proportion of panel in favour of removal	Outcome
Arm bare below cuff	37.5% (3/9)	“The area over which the cuff is placed is of more importance for an automated recording” “Cuff placement over thin clothing does not appear to have significant effect” “Not always possible?”	66%	Consensus not met, therefore retained
Back supported	37.5% (3/9)	“Interpretation and standardization of this difficult”	66%	Consensus not met, therefore retained
Legs uncrossed	12.5% (1/9)	“Unlikely to be practically physiologically relevant”	44%	Consensus not met, therefore retained

Round 2: Following feedback consisting of mean ranks and de-identified comments from Round 1, panellists re-ranked all 10 variables, again with each variable required to receive a unique rank. Round 3: To refine consensus, panellists were presented with five paired comparisons derived from their individual Round 2 rankings. For each pair, panellists made a forced choice, selecting which variable was more important for BP measurement accuracy. This pairwise forced-choice method facilitated final rank stabilization, achieving high consensus (Kendall’s coefficient 0.97, P < 0.001) as detailed in Box 1.

Throughout stage 2, panellists were asked to provide recommendations for additional conditions for an accurate inpatient BP. Several conditions outlined in the guidelines for outpatient measurement were suggested for consideration (Supplementary Tables 5 and 7). These included procedural considerations such as quiet surroundings, repeated measures over time and 5 min of rest. These were not added to the list as the hospital environment is such that ensuring ambient quiet, and rest are not easily replicated. Nor was ensuring the patient is anxiety-free and had no intake of stimulants such as caffeine for a routine BP measurement for similar reasons. The panel advised that a validated device be part of the conditions for an accurate measurement which was felt prudent to consider, as was the possibility of a supine (as opposed to seated) BP protocol be considered given most inpatients are assessed on a bed, rather than a chair and a seated position may not always be medically appropriate (for example post operatively). These latter considerations were brought into the final recommendations.

Note was made in mediated commentary by the panel over the survey that certain unique challenges in the inpatient setting were present that could impact on BP measurement. The panel considered that necessary compensation should be made in how inpatient BPs were interpreted (Supplementary Table 7). The challenges of ranking individual aspects of the procedure of BP measurement for importance was also highlighted, with emphasis on inclusion of all ten key aspects for the BP attained to be reliable.

**Box 1:** Final rank of 10 key variables for accurate inpatient BP measurement as per expert panel consensus (Kendall’s 0.97)

1. At least two BP measurements taken
2. Correct cuff size
3. Not talking during BP measurement
4. Cuff correctly positioned/fitted to upper arm
5. Cuff at heart level
6. Patient in seated position
7. A (validated) device calibrated within recommended period as per manufacturer of device
8. Legs uncrossed
9. Arm bare below cuff
10. Back supported

*NB: listed from most important to least important*

## Discussion

Currently, there are no guidelines for how to accurately measure BP in an inpatient setting. This expert panel have agreed with 100% consensus on the appropriate source documents to guide correct inpatient BP measurement. They took 3 anonymised rounds of ranking-type Delphi surveys to reach a high degree of agreement (Kendall’s coefficient 0.97) on the necessary ten variables for accurate inpatient BP measurement, graded in order of importance for an accurate reading (Box 1). They made two additional recommendations that a validated device be a requirement for inpatient BP measurement, and future consideration of conditions for an accurate supine measurement, given the unique environment of acute hospital care.

This panel highlighted the importance of standardisation of technique and device validation, using internationally recognised standards, for accurate BP measurement. Standardisation of technique is particularly relevant to the acute care setting where many of the patient factors that can alter the accuracy of BP measurement are not controllable such as physiological, pharmacological and environmental inconstancies. To our knowledge, this survey is the first of its kind to provide guidance to hospital-based clinicians on the appropriate components of accurate BP measurement, specific to the acute care population. The components of accurate inpatient measurement have long been recognised as an under researched area with unique contrivances of BP variation^17^ Identification of 10 key variables, ranked for order of importance can now be used as a standard to perform qualitative assessment of routine performance of hospitals in BP measurement and ideally serve as formative steps to develop guidelines to standardise and improve the quality of BP measurement in hospitalised patients. Through better quality BP measurement, diagnosis and treatment of BP variation can improve and achieve better patient outcomes.

The concordance between inpatient BPs and stable office or ambulatory BP readings is unknown and therefore the appropriate response to inpatient measurements for chronic disease management remains in a state of vacillation. Context of BP measurement is known to have a considerable impact on the values measured with the most obvious example of white coat hypertension. Controversies surrounding treatment targets for hypertension have arisen because of the discordance in measured BPs in trials versus routine clinical care. This was well detailed in a linking analysis found a mean difference of 7.3 mmHg in systolic blood pressure between outpatient clinic readings and trial measurements among participants of the SPRINT trial^30^ These variations were not unique to the SPRINT trial, with as many as 6 other studies reaching similar findings, including a variance from ambulatory monitoring^31^ These discrepancies came down to the difference between a standardised measurement technique applied during trials versus the unstructured and variable techniques of routine clinical care. The main purpose of BP measurement is to detect acute clinical deterioration and so the exact accuracy of the readings for more enduring treatment changes and indeed diagnosis of chronic hypertension is problematic. Regardless of the appropriateness of responding to these measurements that is certainly in question, enduring treatment changes are made based on these measurements. Research on treatment intensification for inpatients with elevated BP shows 47% of patients with elevated inpatient BPs were normotensive before admission, leaving room for overdiagnosis, unnecessary prescribing and, at this stage little evidence for cardiovascular benefit^9,10^ The converse is also a concern, where inaccurately ascribing normotension to a hypotensive patient could foreseeably result in increased falls risks as was observed in a 2019 analysis of elderly admitted patients, observing in an increase in 30 day readmission and serious adverse events in individuals with treatment intensification for elevated BPs during a recent hospital admission^10^ These concerns about accuracy of measurement would suggest a significant knowledge gap persists for the appropriate response to BP measurements outside the normal reference range in hospital. Further research, ideally in the form of randomised studies will be required to determine the benefits to treatment changes or other interventions based on hospital BP measurement. A randomised implementation study would also provide a platform to determine the feasibility of some of the variables that may prove challenges in high acuity patients such as adopting a seated position, ideally supported by a controlled validation study of the magnitude of BP effect of being seated in bed versus seated in a chair. This paper provides the basis for a pragmatic guide to targeting accurate readings to inform these studies.

Our panel had 100% retention across all rounds of the survey, a high degree of consensus among a geographically diverse group, good gender representation and a high calibre of expert participation, with most panellists being authors on guidelines informing BP management in the outpatient setting. Note is made of the limitations of the Delphi panel composition, with no representation from low- and middle-income countries, other clinical specialties involved in BP management such as cardiology, and additionally, no nursing or consumer representation. The near-complete concordance achieved in likely reflects in part the pairwise comparison methodology, which facilitated convergence on established measurement principles but should be understood in the context of this methodology and its potential biases. This was a small panel, and a larger number of representatives may have reached a different conclusion, likely over a greater number of rounds of surveys that would have risked loss to follow up. While all panellists were experts in the field of hypertension, not all had the specific professional focus of BP measurement accuracy. Four of the nine expert panellists were co-authors on this study, raising potential concerns about bias. While all panellists remained blinded during the consensus process and met identical expert criteria, we performed a sensitivity analysis excluding co-author responses. This analysis showed minimal impact on results: Kendall’s W coefficient remained high (0.98 vs 0.97), and the top five ranked variables were identical with only minor reordering between positions one and two. These findings suggest that co-author participation did not materially influence the consensus results. Indeed, removing the co-authors actually increased concordance slightly (W = 0.98), indicating they added diversity rather than homogeneity. The main purpose of inpatient BP measurement is to detect acute clinical deterioration and so the exact accuracy of the readings for more enduring treatment changes is problematic. Regardless of the appropriateness of responding to these measurements, enduring treatment changes are made based on these measurements^10^. So while the representativeness of an inpatient BP measurement needs to be better delineated, greater precision in the measurement offers a chance to improve the quality of the informant BP data. This newly formed list of variables has also yet to be compared to directly collected data from observed BP measurements in the hospital setting which means the performance of a representative hospital and the relevance of these parameters has not been interrogated. A pending publication of this study’s authors will examine this in greater detail.

While the output of this Delphi panel is a constructive starting point in the improvement of inpatient BP management, it does not replace the need for empirical research. This is certainly the necessary direction to improve the quality of BP measurement in the acute care setting. The novelty in this case is that this study has taken the first step to assess the veracity of which elements of BP apply to the inpatient setting using a structured methodology and expert opinion by no intention claiming novelty in methodology of BP measurement.

This study identifies 10 key variables affecting BP measurement accuracy in acute care settings, ranked by expert consensus according to their perceived importance. Panellists additionally recommended prioritising validated devices and developing inpatient-specific protocols for supine measurements. These consensus-derived priorities may inform future efforts to standardize routine inpatient BP assessment practices and could serve as a framework for quality improvement initiatives. Further research is needed to validate whether addressing these variables in ranked order improves BP measurement accuracy and patient outcomes in clinical practice.

## Supplementary Information

Supplementary Information.

## Data Availability

All data is provided in this manuscript or its supplementary material. Any additional information can be made publicly available by contacting the corresponding author.
