# Supplementary material for: Factors affecting hospital inpatient blood pressure measurement as ranked by a Delphi survey
Source: Sci Rep. 2026 Apr 5;16:16364. doi: 10.1038/s41598-026-46429-6 (PMC13212961; doi:10.1038/s41598-026-46429-6)

# Appendices

## Document A: Survey one-ratification of source documents (email to panellists)

 Firstly, thank you again for agreeing to participate. This email is the first survey of this project.

The purpose of this panel is to reach a consensus on the ranked order of importance of each component part of measuring a non-invasive inpatient blood pressure in terms of attaining an **accurate** reading.

As you know, there are no current guidelines for the measurement of blood pressure in the inpatient setting. There are however ones for use in the outpatient setting that can be adapted to the hospital environment. Moreover, while there is some evidence for the impact of different variables on the accuracy of blood pressure measurement, it is from a collection of relatively small studies that are insufficient to determine which components of blood pressure measurement are the most important. Thus, the need for this expert panel’s opinion.

Before you undertake the ranking survey, **I would like you to please ratify the selected source documents**. This is a necessary component of a Delphi method study.

These documents outline:

1. The key components of an accurate blood pressure measurement
2. The evidence for which patient, procedure and observer elements have the greatest impact on the accuracy of the results obtained.

Please submit your responses by **XXX**

1. **Key Source documents for blood pressure measurement**

Do you agree the following documents are appropriate to guide the necessary components of accurate blood pressure measurement? (attached above or hyperlinked below for reference)

[The Management of Elevated Blood Pressure in the Acute Care Setting: A Scientific Statement from the American Heart Association](https://pubmed.ncbi.nlm.nih.gov/38804130/)

[International Consensus on Standardized Clinic Blood Pressure Measurement**-A Call to Action**](https://pubmed.ncbi.nlm.nih.gov/36621637/)

[Measurement of Blood Pressure in Humans: A Scientific Statement from the American Heart Association](https://pubmed.ncbi.nlm.nih.gov/30827125/)

[The 2020 “WHO Technical Specifications for Automated Non-invasive Blood Pressure Measuring Devices with Cuff”](https://pubmed.ncbi.nlm.nih.gov/33517681/)

Do you agree with the above list of source documents? Please respond yes or no below. If not, please indicate why.

|  |
| --- |

Are there any additional source documents that you think ought to be considered for accurate inpatient blood pressure measurement components? Please detail them here.

|  |
| --- |

1. **Source document for sources of inaccuracy in inpatient blood pressure measurement**

Do you agree that the following systematic review is an appropriate guide to the magnitude of impact of key variables on the accuracy of blood pressure measurement? (attached above or hyperlinked below for reference)

[**Sources of inaccuracy in the measurement of adult patients’ resting blood pressure in clinical settings: a systematic review**](https://pubmed.ncbi.nlm.nih.gov/27977471/)

Please state whether you agree or disagree. Please state why not if you disagree and feel free to add any other relevant comments.

|  |
| --- |

Do you recommend any additional source documents for determining the magnitude of impact each component of blood pressure measurement has on accuracy?

|  |
| --- |

Do you have any additional comments you would like to make about important source documents for determining the important component parts of accurate inpatient blood pressure assessment?

|  |
| --- |

Thank you so much for your participation. Please do not hesitate to contact me if you have any questions.

## Document B: Qualtrics Survey-Inpatient blood pressure measurement ranking of variables round one

Thank you again for agreeing to participate in this project. The purpose of this survey is to rank key components of blood pressure measurement order of importance for **accuracy** in an inpatient setting. 

To help you in the task of ranking these component parts of a blood pressure measurement, **you have been sent a summary table** from the systematic review detailing the evidence the magnitude of effect each procedural aspect potentially has on the accuracy of the obtained blood pressure.

1.
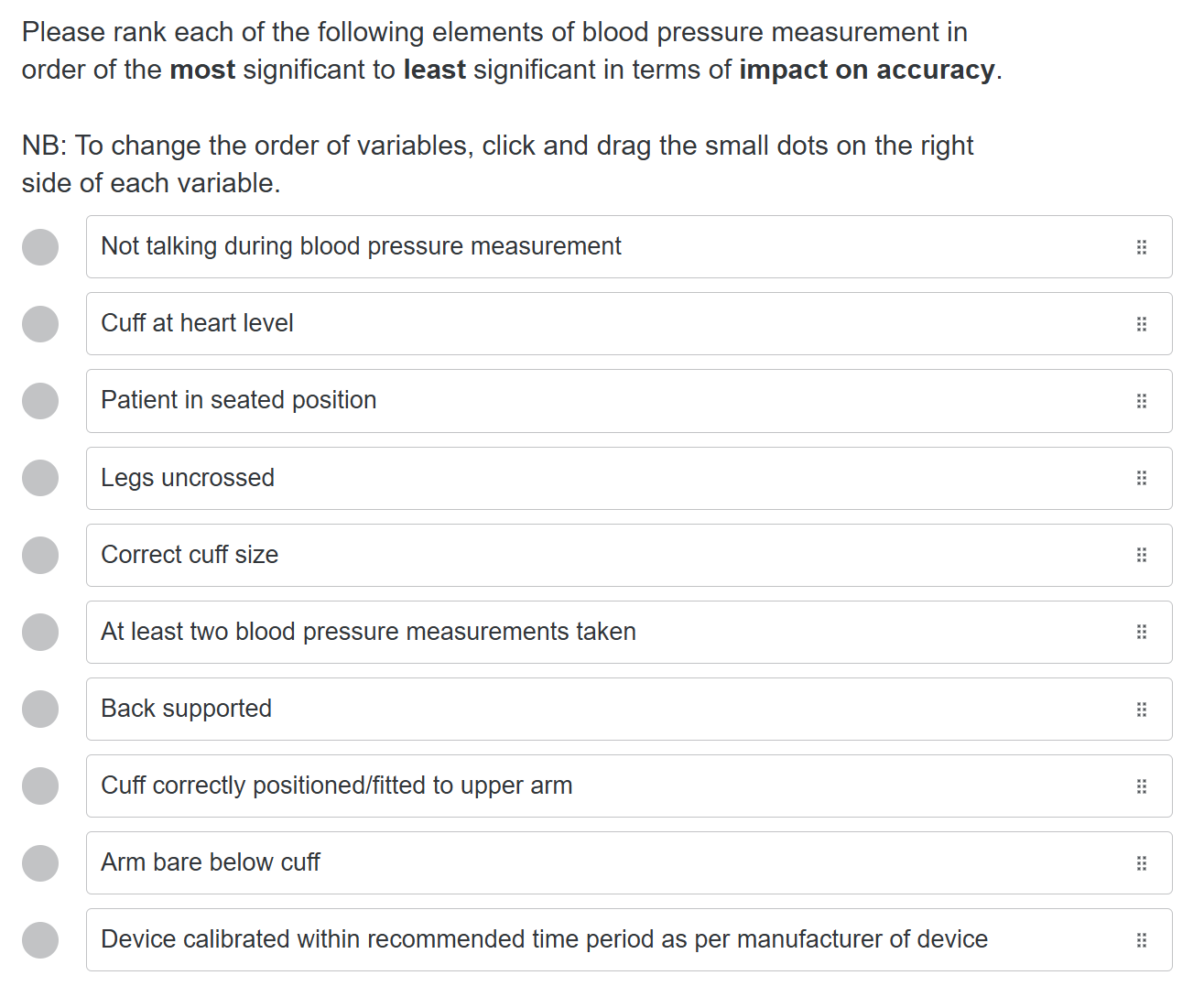

2. (Optional) This section will allow you to comment on the necessity of the selected variables. Please leave blank if you agree all should be included.

   Do you feel any of the variables **are not**essential to an accurate blood pressure?


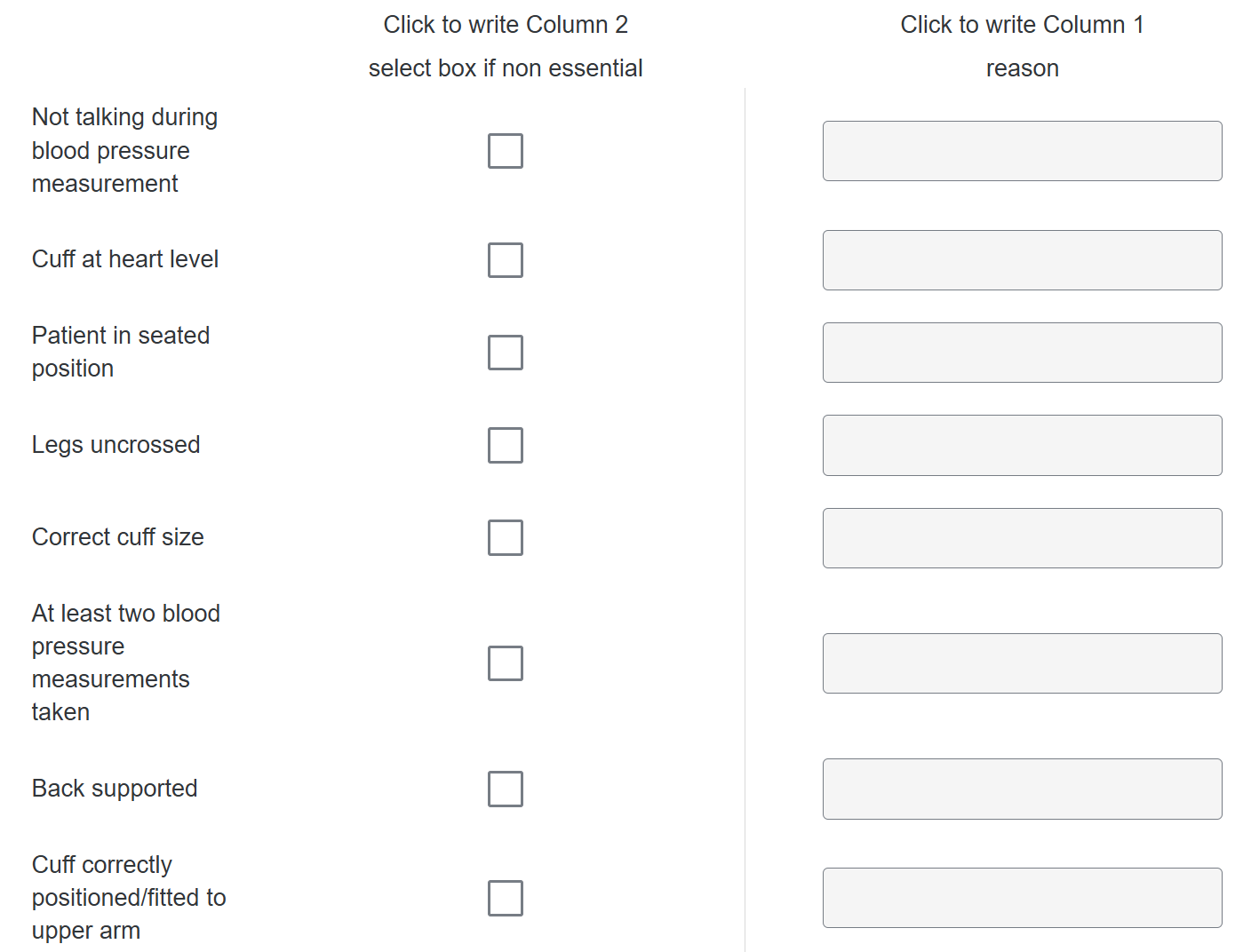

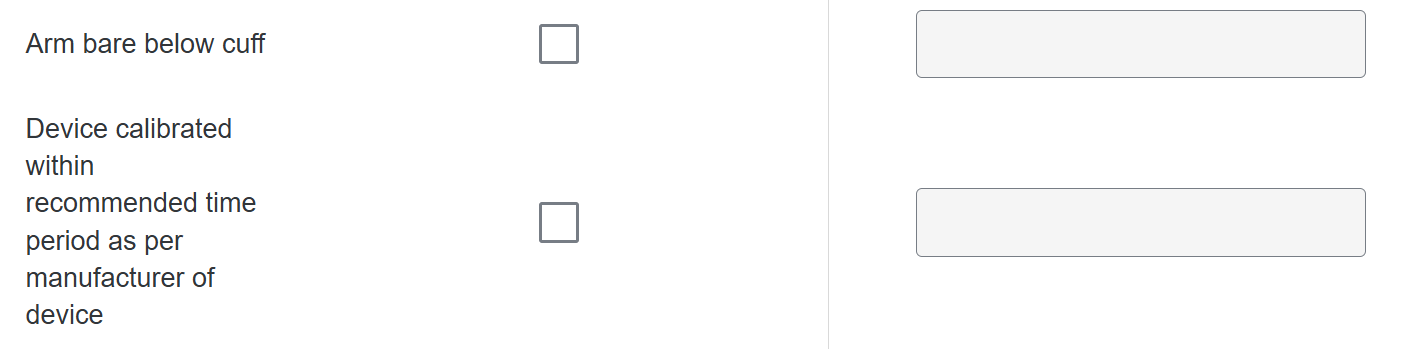


1. Are there any additional elements of blood pressure measurement that **should** be included in the composite measurement not already listed? If so, please detail them here and provide a brief rationale as to why.

|  |
| --- |

## Document C: Qualtrics survey- Inpatient blood pressure measurement ranking of variables round two

As a reminder, the purpose of this survey is to rank key components of blood pressure measurement order of importance for **accuracy** in an inpatient setting. 

Consensus on the order of importance of each of the variables has not yet been met. The mean rank from survey one has been added next to each variable. Additionally, some of the variables have been nominated for removal from the list.

**Please use the provided summary**of survey one emailed to you to inform your responses in this second round. You may also like to refer back the summary table from the systematic review which was supplied in survey one and also sent again in your survey link email.

1. Please rank each of the following elements of blood pressure measurement in order of the **most** significant to **least** significant in terms of **impact on accuracy**. **Mean rank** from survey one is provided next to each variable.

   NB: To change the order of variables, click and drag the small dots on the right side of each variable.


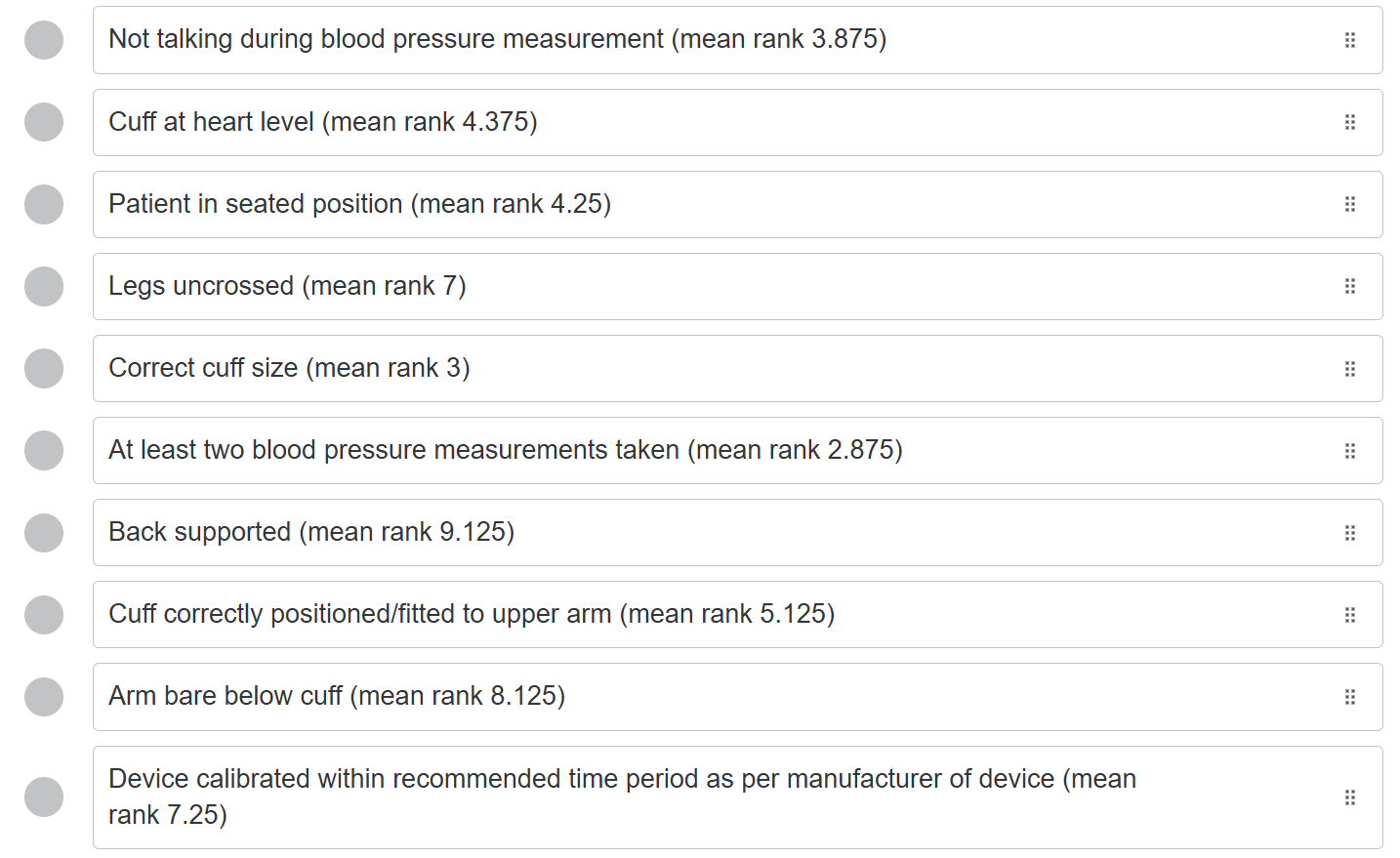


2.
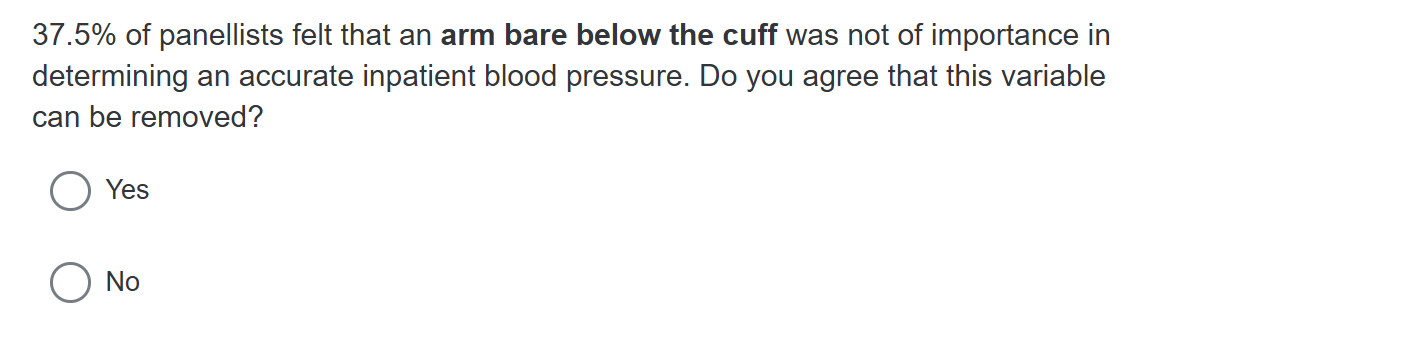


3.
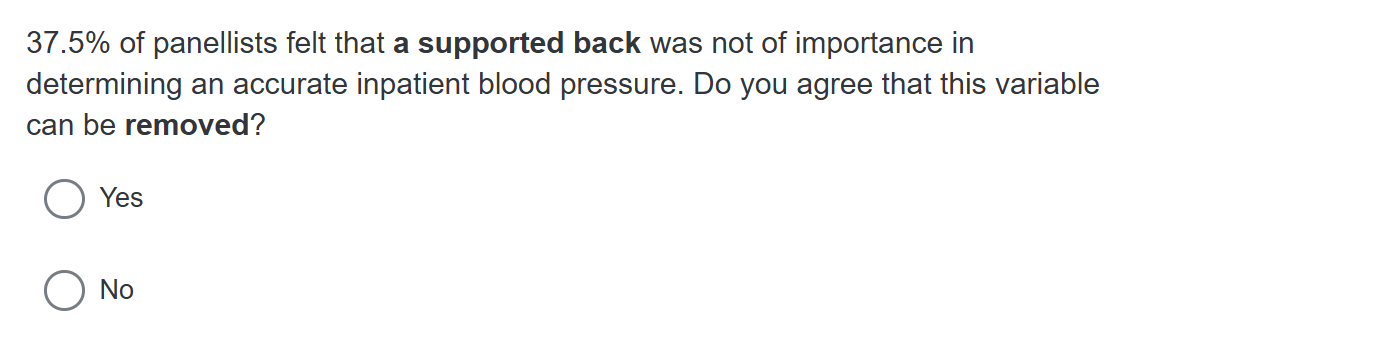


4.
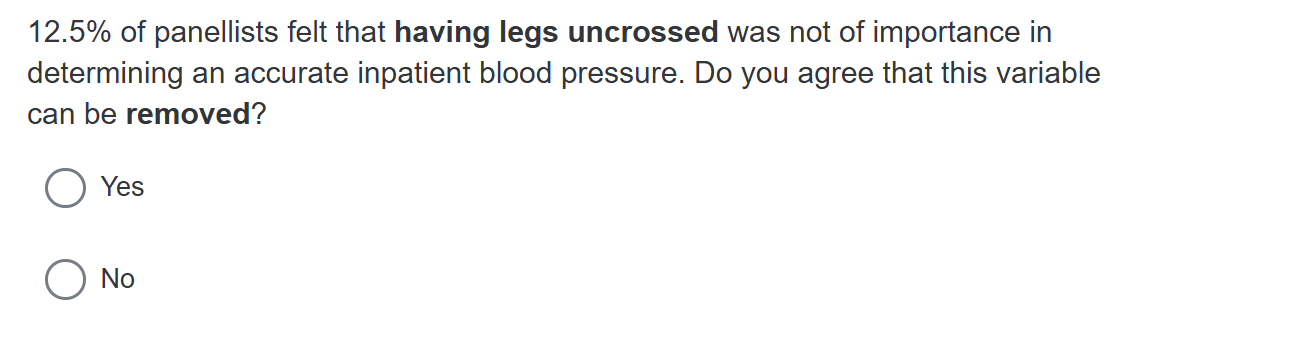


5.
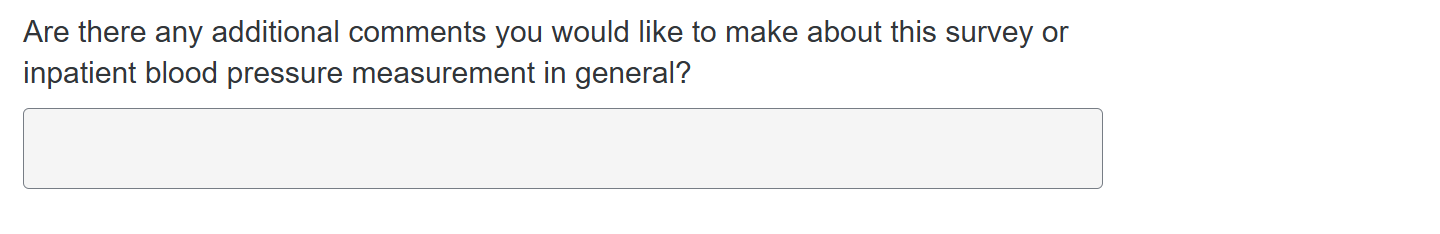


## Document D: Qualtrics survey- Inpatient blood pressure measurement ranking of variables round three

In the last two rounds of this survey, the approximate order of importance of each variable has been determined from most important for accuracy, to least important for accuracy of inpatient blood pressure assessment (where a score of 1 means most important and a score of 10 means least important). 

The final order of each variable must now be determined by reviewing each pair of closely ranked variable for their final order. Please indicate which of each paired variable you feel is most important for accuracy in the questions below.

1.
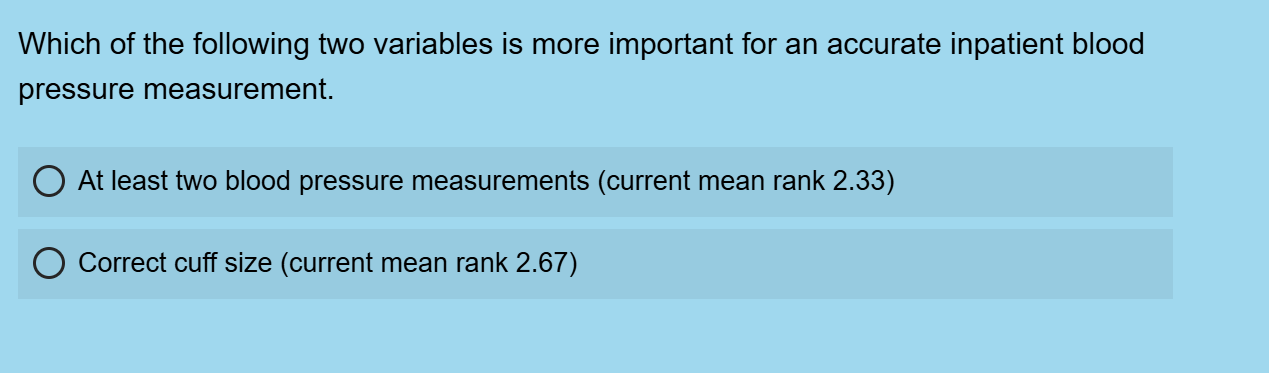


2.
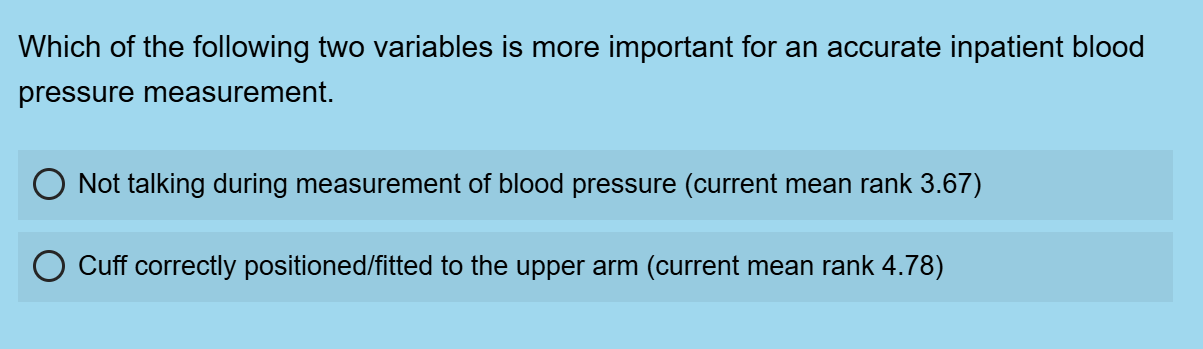


3.
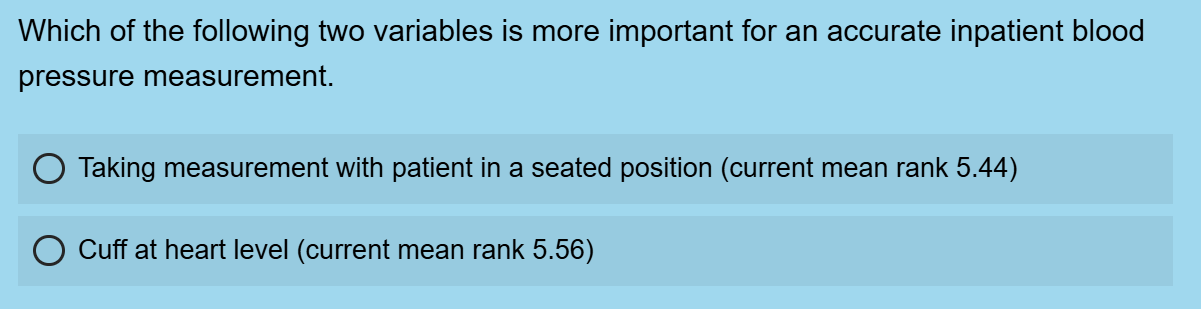


4.
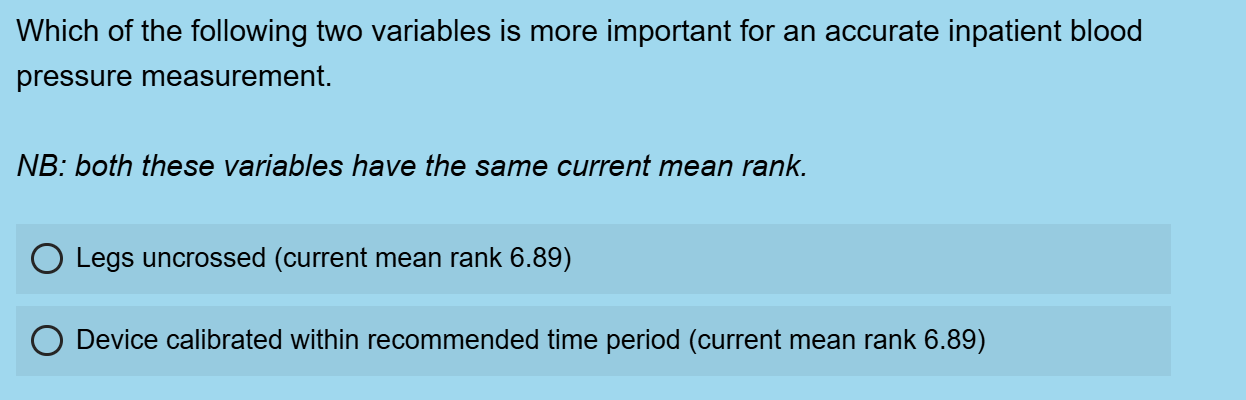


5.
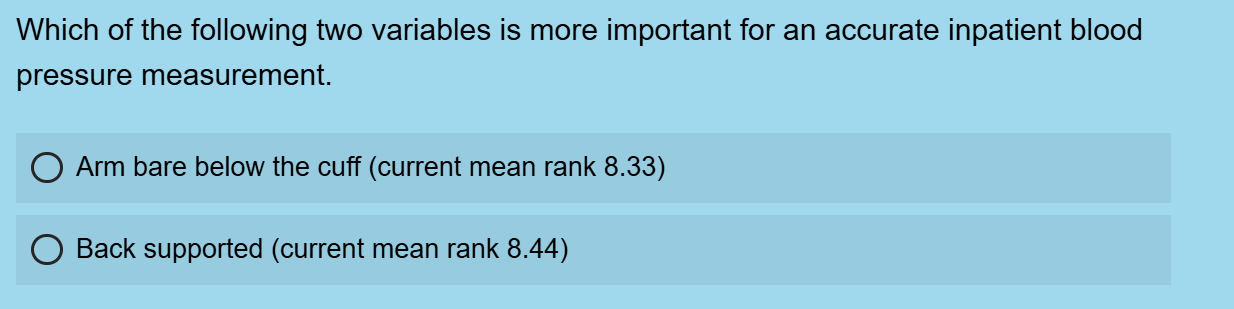


## Supplementary table 1: Search strategy for blood pressure measurement guidelines

| **Objective:** identify what evidence exists (1) to guide accurate measurement of BP, regardless of environmental setting, (2) to guide accurate measurement of BP in the inpatient setting and (3) to determine which aspects of BP measurement has the greatest magnitude of effect on accuracy |
| --- |
| **Time period:** January 1, 2014-May 31, 2024  **Sources:** Advanced Google search, direct search of professional society websites and review of cited texts in identified sources.  **Inclusion criteria:** Guidelines from national or international professional societies with focus on blood pressure measurement techniques.  NB: if more than one guideline was available from a given society of nation, the most current guideline only was included, so long as there was no major difference in the scope of the guideline. If the latter, both were included. |
| **Advanced Google search string:** (“blood pressure” OR “hypertension” OR “blood pressure measure*”) AND (“guideline*” OR “recommendation*” OR “consensus statement”) AND (“measure*”)  **NB:** The first 10 pages (100 results were screened) for relevance |
| **Directly searched professional societies:**   1. American College of Cardiology (<https://www.acc.org/>) 2. American Heart association (https://www.heart.org/) 3. European Society of Cardiology (https://www.escardio.org/) 4. European Society of Hypertension (https://www.eshonline.org/) 5. International Society of Hypertension (https://ish-world.com) 6. National Heart Foundation of Australia (https://www.heartfoundation.org.au/for-professionals/hypertension) 7. Hypertension Canada (https://hypertension.ca/) 8. NICE UK (https://www.nice.org.uk/guidance/ng136) 9. World Health Organisation (https://www.who.int/publications/i/item/9789240002654) |

## Supplementary table 2: Summary table of magnitude of effects of sources of inaccuracy relevant to inpatient blood pressure adapted from Kallioinen et al.^(16)^

|  | **Range of reported significant mean effects (mmHg)** | |  |  |  |  |
| --- | --- | --- | --- | --- | --- | --- |
| **Variable/ Condition** | **SBP mean effects** | **DBP mean effects** | **comparison** | **narrative description** | **Number of studies** |  |
| Talking during measurement | 4 to 19 | 5 to 14.3 | compared to not talking | Small-large SBP effects, small-moderate DBP effects | 6 |  |
| Arm lower than heart level | 3.7 to 23 | 2.8 to 12 | compared to at heart level | small to large effects for both SBP and DBP | 10 |  |
| Body position | | | | | 9 |  |
| *standing* | -2.9 to 5 | 7 | compared to seated | small to moderate effects |  |  |
| *supine* | -10.7 to 9.5 | -13.4 to 6.4 | compared to seated | small to moderate effects |  |  |
| Legs crossed at knees | 2.5 to 14.89 | 1.4 to 10.81 | compared to feet flat on floor | small to moderate effects | 7 |  |
| Incorrect cuff size | | | | | 6 |  |
| *too small* | 2.08 to 11.2 | 1.61 to 6.6 | compared to correct cuff size | small to moderate effects |  |  |
| *too large* | -3.7 to -1.45 | -4.7 to -0.96 |  | small effects |  |  |
| Reliance on a single measurement | 3.3 to 10.4 | -2.4 to 0.6 | compared to ≥2 consecutive measures | 1st reading is small to moderately higher | 6 |  |
| Unsupported back | nil effects | 6.5 | compared to supported | DBP moderate effects | 1 |  |
| Clothing under cuff | not significant | not significant | compared to bare arm | not thought to have significant effect | 4 |  |
| Ill fitted cuff | NB: not in systematic review but recommended by expert consensus guidelines. | | | n/a | n/a |  |
|  |  |  |  |  |  |  |
|  |  |  |  |  |  |  |
| Device calibration error | | | | | 3 aneroid, >40 automated |  |
| *aneroid device* | 1.4 to 69.7% of individual devices >3mmHg calibration error | | | A theoretical risk of poor calibration if recommended calibration period has lapsed |  |  |
| *automated device* | 4.5 to 26% of individual devices >3mmHg calibration error | | |  |  |  |

*NB: size of potential inaccuracies in narrative description: small: <5mmHg, moderate 5-15mmHg, large >15mmHg*

## Supplementary table 3: Key source documents ratified by expert panel

| **Supplementary Table 3: Key source documents ratified by expert panel** | | | |
| --- | --- | --- | --- |
| **Authors (year)** | **Society** | **Title** | **Consensus** |
| *Representative guidelines* | | | |
| Bress et al. (2024)^(17)^ | AHA | Management of Elevated Blood Pressure in the Acute Care Setting: A Scientific Statement from the American Heart Association | 100% indicated “yes” as appropriate |
| Cheung et al. (2023)^(4)^ | n/a | International Consensus on Standardized Clinic Blood Pressure Measurement - A Call to Action |  |
| Muntner et al. (2019)^(3)^ | AHA | Measurement of Blood Pressure in Humans: A Scientific Statement from the American Heart Association |  |
| John et al. (2021)^(19)^ | WHO | The 2020 "WHO Technical Specifications for Automated Non-Invasive Blood Pressure Measuring Devices with Cuff" |  |
| *Systematic review* | | | |
| Kallioinen et al. (2017)^(16)^ | n/a | Sources of inaccuracy in the measurement of adult  patients’ resting blood pressure in clinical settings:  a systematic review | 100% indicated “yes” as appropriate |

*AHA: American Heart Association, WHO; World Health Organisation, n/a: nonapplicable*

## Supplementary table 4: Inpatient Blood Pressure Measurement Expert Panel

| Name | Location | Gender | Expertise |
| --- | --- | --- | --- |
| Mark Brown | NSW, Australia | Male | Nephrologist: clinician/researcher |
| Louise Burrell | VIC, Australia | Female | Researcher |
| Swapnil Hiremath | Ottawa, Canada | Male | Nephrologist: clinician/researcher |
| Sradha Kotwal | NSW, Australia | Female | Nephrologist: clinician/researcher |
| Adeera Levin | British Columbia, Canada | Female | Nephrologist: clinician/researcher |
| Angela Makris | NSW, Australia | Female | Nephrologist: clinician/researcher |
| George Mangos | NSW, Australia | Male | Nephrologist: clinician/researcher |
| Aletta E Schutte | NSW, Australia | Female | Researcher |
| James Sharman | TAS, Australia | Male | Researcher |

## Supplementary table 5: Additional panellist responses to stage one of Delphi survey

|  | **Panellist Comments** |
| --- | --- |
| Do you agree the proposed four documents are appropriate representative papers to guide the necessary components of accurate blood pressure measurement? | - Yes- good set of overarching documents – but only focusses on adults. This set would be improved by including similar documents on children esp. normal cut off values and pregnancy - Yes, they are all relevant. I would suggest we emphasise that this is NOT for pregnancy; otherwise, I’d suggest adding the ISSHP Guidelines - Yes agree – although confirming CoI – where I have co-authored two of these (WHO Technical specifications and the International Consensus document) |
| Do you agree that the provided systematic review by Kallioinen et al. is an appropriate guide to the magnitude of impact of key variables on the accuracy of blood pressure measurement? | - Nice summary of data. I have an issue with clothes as it depends on what you call clothes- the puffer jacket and 3 layers may affect measurements as opposed to a light short or single thin layer – Otherwise ideal - Very thorough review of available papers and consideration of potential confounding influences on BP measurement - This is a very detailed analysis; probably unique. Very relevant for non-inpatient BP measures but doesn’t clearly separate studies of in-hospital measurement (I don’t think they had any?). - Table 2 is very helpful (and informative in that bi-directional errors occurred for many variables) - The Kallioinen review is well-known for listing the various contributing factors based on a systematic review. Excellent to include that. - Agree that this paper provides a reasonable summary of the available evidence. However, the analysis has significant limitations, and the quality of the evidence is variable. - Agree with this overview and serves as a good base |
|  | |
| Comments on additional resources for determining the magnitude of impact each component of blood pressure measurement has on accuracy | - Not aware of another. - not sure that people would read individual articles unless very interested in topic, the SR serves as a good base |
| General additional comments | - Would just add information on kids and pregnancy similar to the information you have above I am not aware of any specific kid and pregnancy documents that go into that level- rather gleaned from other documents i.e. appropriately validated machines, auscultate in kids then oscillometric. - Arm circumference itself is not included as a potential for error, as far as I can see.  The larger the arm the more difficult it may be to fit a cuff, especially with very large arms, even if the cuff is longer like a thigh cuff.  At that size, one wonders whether the relationship between direct BP measurement and indirect measurement still exists. - There is a significant difference between validation and calibration for any automated device and as clinicians we must decide whether we accept a device that’s calibrated even if not validated. It’s worth putting this issue front and centre. - important to synthesize the harm and variability of current approaches in inpatient BP treatment practices more fully so that people appreciate the problem more...especially targeting for example teaching hospitals |

## Supplementary table 6: Panellist-recommended resources during stage one for consideration of the variables impacting on accuracy of inpatient BP measurement

| **Panellist-recommended additional resource** | **Panellist comment** |
| --- | --- |
| *Additional resources to guide the necessary components of accurate blood pressure measurement* | |
| Whelton PK et al. 2017 ACC/AHA/AAPA/ABC/ACPM/AGS/APhA/ASH/ASPC/NMA/PCNA Guideline for the Prevention, Detection, Evaluation, and Management of High Blood Pressure in Adults: A Report of the American College of Cardiology/American Heart Association Task Force on Clinical Practice Guidelines. J Am Coll Cardiol. 2018;71(19): e127-e248. | This paper has been widely used (from ACC/AHA etc) – perhaps overtaken by the recent AHA guideline but has a large section on (non-inpatient) BP methodology. So, it's not solely or exactly relevant to inpatient BP but neither are the other documents (panellist 1) |
| Unger et al. 2020 International Society of Hypertension Global Hypertension Practice Guidelines. Hypertension. 2020; 75:1334-1357. DOI: 10.1161/HYPERTENSIONAHA.120.15026 | Guidelines for HTN with global audience. (panellist 2) |
| Schutte et al. Blood pressure and its variability: classic and novel measurement techniques. Nature reviews Cardiology. Oct 2022 | Insights into the comparative values of variable measures of BP, especially BPV (panellist 3) |
| Stergiou et al. European Society of Hypertension recommendations for the validation of cuffless blood pressure measuring devices: European Society of Hypertension Working Group on Blood Pressure Monitoring and Cardiovascular Variability. J Hypertension. 2023 Dec 1;41(12):2074-2087.   doi: 10.1097/HJH.0000000000003483. Epub 2023 Jun 22. | The European Society of Hypertension Working group on BP measurement and variability is perhaps the leading global body with several position statements on accurate BP measurement (panellist 3) |
| *Additional source documents for determining the magnitude of impact each component of blood pressure measurement has on accuracy* | |
| Ishigami, J et al. Effects of Cuff Size on the Accuracy of Blood Pressure Readings: The Cuff (SZ) Randomized Crossover Trial. JAMA Internal Medicine. 2023;183(10):1061-1068 | Provided by panellist 4 |
| Brady, TM et al. Effects of Different Rest Period Durations Prior to Blood Pressure Measurement: The Best Rest Trial. Hypertension. 2021;78(5):1511-1519. | Suggest rest periods of <5 minutes may be as accurate |
| Liu, H., Zhao, D., Sabit, A., Pathiravasan, C.H., Ishigami, J., Charleston, J., Miller, E.R., Matsushita, K., Appel, L.J. and Brady, T.M., 2024. Arm Position and Blood Pressure Readings: The ARMS Crossover Randomized Clinical Trial. *JAMA Internal Medicine*. | Provided by panellist 1 |
| *Additional resources provided in support of general comments* | |
| Stergiou GS, Alpert B, Mieke S, Asmar R, Atkins N, Eckert S, et al. A Universal Standard for the Validation of Blood Pressure Measuring Devices. Hypertension. 2018;71(3):368-74. | Appropriate methods of device validation (panellist 1) |
| Sharman JE, O'Brien E, Alpert B, Schutte AE, Delles C, Hecht Olsen M, et al. Lancet Commission on Hypertension group position statement on the global improvement of accuracy standards for devices that measure blood pressure. Journal of hypertension. 2020;38(1):21-9. | Highlights importance of standardisation of device validation (panellist 1) |
| Armitage, L.C., Mahdi, A., Lawson, B.K., Roman, C., Fanshawe, T., Tarassenko, L., Farmer, A.J. and Watkinson, P.J., 2019. Screening for Hypertension in the INpatient Environment (SHINE): a protocol for a prospective study of diagnostic accuracy among adult hospital patients. *BMJ open*, *9*(12), p. e033792. | Protocol for upcoming randomised trial (panellist 2) |
| Falkner, B., Gidding, S.S., Baker-Smith, C.M., Brady, T.M., Flynn, J.T., Malle, L.M., South, A.M., Tran, A.H. and Urbina, E.M., 2023. Pediatric primary hypertension: an underrecognized condition: a scientific statement from the American Heart Association. *Hypertension*, *80*(6), pp. e101-e111. | Provided by panellist 5 in support of need for paediatric and pregnancy specific guidelines |
| Kho CL, Brown MA, Ong SLH, Mangos GJ. Blood pressure measurement in pregnancy: the effect of arm circumference and sphygmomanometer cuff size. Obstetric Medicine. 2009;2(3):116-120. doi:10.1258/om.2009.090017 | Provided by panellist 6 in support of comments on arm size affecting cuff fit |

## Supplementary table 7: General comments from panellists from Qualtrics survey round one

| **Additional elements of blood pressure measurement that should be included in the composite measurement not already listed** | - Repeated measures over time to make the best assessment of blood pressure i.e. one high reading - even if well done doesn't make a diagnosis e.g. may be affected by lack of sleep etc, ideally a quiet room also, free from recent stimulants e.g. no recent energy drink.... (panellist 7) - Use of a Validated device, not just a calibrated device (panellist 1) - Anxiety level of patient. I have found that BP at the start of the appointment is often high and settles over the course of the conversation (panellist 2) - empty bladder; no coffee or smoking prior; at least 5 minutes resting period (panellist 7) - In an inpatient setting it may not always be practical to measure in the seated position... a supine BP measurement protocol may need to be considered separate from the standard seated protocol (panellist 4) - quiet environment- i.e. not taking BP at a sausage sizzle (panellist 8) |
| --- | --- |
| **Additional comments about this survey or inpatient blood pressure measurement in general** | - Perhaps a discussion about it being OK to accept greater error in inpatient measurement technique than for outpatients because less nuanced treatment is required, or advised, during hospital stay (panellist 1). - For inpatient BP, it is not clear that the same rules should apply as for outpatient BP. Inpatient BP is measured as a vital sign and not for management of BP for CV risk. Hence - beyond cuff size etc, the other factors are less important. (panellist 7) - It is difficult to rank order the magnitude of effect for individual components of BP measurement (on accuracy) when a standardised approach that includes adherence to ALL components of the BP measurement protocol is optimal for valid BP readings to be acquired (panellist 4) |

## Figure 1: PRISMA flow diagram of guideline identification and selection


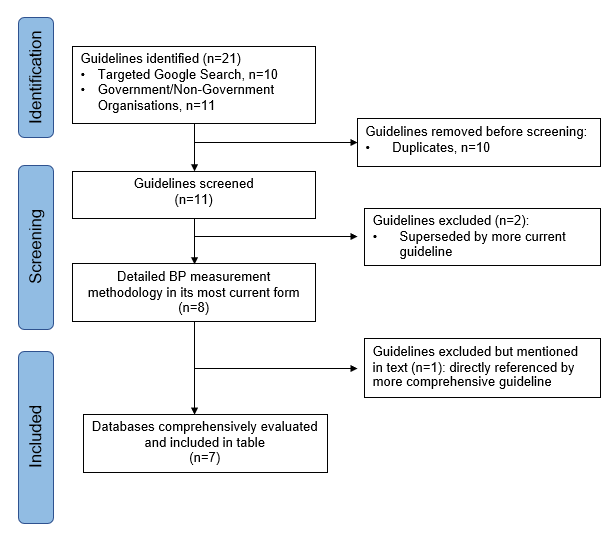

Supplement: Supplementary file 1 — Supplementary Information. [file 41598_2026_46429_MOESM1_ESM.docx]
